# RETRACTION: “LINC02273 Promotes Hepatocellular Carcinoma Progression via Retaining *β*‐Catenin in the Nucleus to Augment Wnt Signaling”

**DOI:** 10.1155/bmri/9820432

**Published:** 2026-05-21

**Authors:** BioMed Research International

RETRACTION: L. Peng, R. Ye, X. Zhu, Y. Xie, B. Zhong, Y. Liu, H. Li, B. Xie, “LINC02273 Promotes Hepatocellular Carcinoma Progression via Retaining *β*‐Catenin in the Nucleus to Augment Wnt Signaling,” BioMed Research International, 2022, 9631036, https://doi.org/10.1155/2022/9631036

The above article, published online on 28 January 2022 in Wiley Online Library (wileyonlinelibrary.com), has been retracted by agreement between journal Editor‐in‐Chief, Yoel Klug; and John Wiley & Sons Ltd. The retraction has been agreed due to concerns raised with Figure 2e on PubPeer [1]. Specifically, the Hep3B/NT panel contains repeated elements, when compared to the Sh#2 panel in Figure 4e of the work of a different author group [2]. The images include rotations and additional field of view which indicate that the data were derived from the same image, though each article describes different experimental conditions.

The authors responded to an inquiry by the publisher, but their explanation and the original data provided did not adequately explain how data had been shared between different author groups. The retraction has been agreed to because the evidence of data shared between the articles and the lack of a sufficient explanation has compromised the editors’ confidence that the results presented in this article are accurate.

The authors agree to the retraction.
